# Back to the future: malabsorption is the Achilles’ heel of hypoabsorptive metabolic/bariatric procedures

**DOI:** 10.1093/bjs/znae073

**Published:** 2024-03-29

**Authors:** Ricardo V Cohen, Paulina Salminen, Philip R Schauer, Francesco Rubino

**Affiliations:** Centre for Obesity and Diabetes, Oswaldo Cruz German Hospital, Sao Paulo, Brazil; Division of Digestive Surgery and Urology, Turku University Hospital, Turku, Finland; Department of Surgery, University of Turku, Turku, Finland; Metamor Institute, Pennington Biomedical Research Center, Baton Rouge, LA, USA; Bariatric and Metabolic Surgery, King’s College Hospital, London, UK

Papadia *et al*.^1^ have reported 30-year follow-up of the classic hypoabsorptive open Scopinaro biliopancreatic diversion (S-BPD). Perioperative mortality was higher than today’s rate, mainly because, currently, a standardized preoperative risk assessment and the laparoscopic approach can mitigate the negative operative issues involved in major abdominal operations in people with obesity and its complications. Long-term follow-up data were available for 61% of patients at 20 years and 19% (38 of 199) at 30 years; most surgery-related complications decreased over time and were generally related to the laparotomic access. Despite the excellent weight loss and metabolic outcomes, markedly the effects over type 2 diabetes (T2D), nutritional issues were an ongoing problem during late follow-up of S-BPD. Similarly, Mingrone *et al*.^2^ reported good outcomes in a 10-year follow-up of an RCT that involved S-BPD and Roux-en-Y gastric bypass (RYGB). The significance of the nutritional side-effects of S-BPD was already well known. Even in a small trial, patients had significantly more nutritional complications after S-BPD than RYGB, which had a better risk–benefit safety profile.

The safety issues of this operation, however, cannot be extrapolated to modern bariatric surgery, especially the most commonly performed procedures such as RYGB and sleeve gastrectomy (SG), whose remarkably safe profile has been verified in numerous studies, including multicentre series and RCTs^3^.

The study by Papadia *et al*. has several limitations, as it reflects a single-centre practice, and follow-up data beyond 20 years were only available for less than 20% of the original patients. However, the observations arguably derive from one of the best centres for BPD-S, and very few studies have follow-up data for any procedure beyond 20 years. Thus, the study provides a unique opportunity to assess the safety and efficacy of hypoabsorptive procedures such as BPD-S.

The data presented in this paper confirm the remarkable efficacy of S-BPD, but also prove, beyond any reasonable doubt, that nutritional complications remain the Achilles’ heel of this operation.

Nevertheless, different operative proposals based on intestinal hypoabsorption have emerged. The one-anastomosis gastric bypass (OAGB), single-anastomosis duodenoileal bypass plus SG (SADI-S), or even procedures such as transit bipartition, are innovative operations that have mostly short-term reports of efficacy with scarce data on safety^4^. According to the American Metabolic and Bariatric Surgery Quality Improvement Program database^5^, SADI-S had higher operative complication rates than RYGB. Furthermore, the only case series^6^ published on SADI-S with 10 years of follow-up reported a reoperation rate of 7.5% owing to refractory hypoproteinaemia.

In several different RCTs, OAGB was not superior to RYGB in terms of weight loss and control of obesity complications. However, in studies with 2 years or more of follow-up, besides bile reflux, nutritional problems were significantly more common than after RYGB.

The other proposals, such as transit bipartition and its single-anastomosis version, have short-term efficacy data and no nutritional problems. This is probably because the case series published usually had 12 months of follow-up, and macronutrient and micronutrient deficiencies tend to appear during longer follow-up.

No published study on innovative procedures has provided data other than those related to weight loss and sometimes glycaemic control. For example, most did not assess cardiovascular risk factors or renal endpoints. Hypoabsortive procedures may lead to better long-term weight loss, as recently shown with the duodenal switch (DS), another hypoabsorptive technique.

A recent study showed that DS^7^ (and SADI-S) produced greater weight loss at 24 months than RYGB and SG (40.6 *versus* 33.8 and 28.5% respectively; *P* < 0.001). However, the rate of control of the co-morbidities analysed (T2D, hypertension, and hyperlipidaemia) was similar.

There is evidence that the magnitude of weight loss does not have a linear correlation with metabolic control^8^. Total weight loss (TWL) greater than 25% may not confer additional metabolic benefits. The lack of significant co-morbidity resolution attenuates the clinical importance of greater TWL after hypoabsorptive procedures. Thus, the balance of risk to benefit of hypoabsorptive procedures seems difficult to justify in many or most patients with obesity and T2D. The greater potential weight loss of S-BPD and other hypoabsorptive procedures in patients with higher BMI has historically justified the preferential use of such procedures in patients with higher BMIs. This rationale is likely to be questioned in an era where new obesity management medications can be used to further the weight loss outcomes of safer procedures, such as RYGB and SG^9^.

Papadia *et al*. remind us that operations should be judged for their long-term effects and that novel procedures should be compared with well tested standard operations like RYGB before they are used in routine clinical practice. Secondary to this lack of robust data on longer-term efficacy and safety, the International Federation for the Surgery of Obesity and Metabolic Disorders^10^ issued a position statement on innovations in metabolic and bariatric surgery, and considered that all such interventions need strict ethical supervision until long term quality data on safety are available.
